# Long‐Term Response of Equids With Pituitary Pars Intermedia Dysfunction to Treatment With Pergolide

**DOI:** 10.1111/jvim.70109

**Published:** 2025-05-02

**Authors:** Harold C. Schott, Julie R. Strachota, Judith V. Marteniuk, Kent R. Refsal

**Affiliations:** ^1^ Department of Large Animal Clinical Sciences Michigan State University East Lansing Michigan USA; ^2^ Veterinary Diagnostic Laboratory, College of Veterinary Medicine Michigan State University East Lansing Michigan USA

**Keywords:** adrenocorticotropin, cortisol, Cushing's disease, horse, insulin, laminitis, survival

## Abstract

**Background:**

Limited data document long‐term responses of equids with pituitary pars intermedia dysfunction (PPID) to pergolide treatment.

**Objectives:**

Report clinical response, medical problems, outcome, and owner satisfaction with pergolide treatment of PPID‐affected equids over 14 years.

**Animals:**

Thirty client‐owned equids with PPID.

**Methods:**

After completion of an open field clinical efficacy study for Prascend® (pergolide tablets), 28 horses and two ponies were enrolled in an extended treatment study (13 receiving 2 μg/kg PO q24h and 17 receiving 4 μg/kg PO q24h). Clients were interviewed every 3 months and equids were re‐evaluated after 2.5, 3, 3.5, 4.5, 5.5, 6.5, 9.5, 12.5, and 14.5 years of treatment.

**Results:**

Five equids died and 24 were euthanized (five for chronic laminitis) during the study period (median survival time, 3.6 years; range 0.6–10.5 years). Seven of 13 equids had a dosage increase to 4 μg/kg PO q24h (maximum study dose) between 1.7 to 4.7 years of the study. After 5.5 years, owners of 13 surviving equids reported sustained clinical improvement and endocrine test results normalized in 75%. After 9.5 years of treatment, only two of six surviving equids had normal endocrine test results.

**Conclusions and Clinical Importance:**

Long‐term treatment of PPID‐affected equids with pergolide produces clinical improvement in nearly all affected animals and normalization of endocrine test results in some cases. Furthermore, this extended treatment study determined that equids can respond favorably long‐term to the initial pergolide dose, rather than needing a progressive increase in dose over time.

**Abbreviations**
ACTH: adrenocorticotropin
CGIT: combined insulin and glucose tolerance test
ID: insulin dysregulation
IRMA: immunoradiometric assay
ODST: overnight dexamethasone suppression test
PPID: pituitary pars intermedia dysfunction
RIA: radioimmunoassay
UK: United Kingdom

## 1 Introduction

United States Department of Agriculture studies indicate an increase in equids aged 20–29 years from 7.6% to 11.4% of the total equine population between 2005 and 2015 [1]. Similar surveys in the United Kingdom (UK) and Australia indicated that 29% and 33% of equids were 15 years or older, respectively, with 2.2% of the UK equine population > 30 years old [2, 3].

A consequence of an aging population of equids is increased diagnosis of medical problems, including osteoarthritis, asthma, neoplasia, and endocrinopathies [4]. Pituitary pars intermedia dysfunction (PPID) is the most commonly recognized endocrinopathy of aged equids, with a prevalence of 2.9% in a population of 70 000 equids in seven UK veterinary practices [4]. Clinical signs of PPID, specifically hair coat changes, have been reported in > 20% of equids ≥ 15 years of age in Australia and the UK, increasing to > 30% for equids over 30 years of age [3, 5, 6]. However, the number of equids recognized as having PPID by owners in these studies was < 5%, and < 70% of equids with owner‐recognized PPID were receiving treatment for PPID [5, 6]. Similarly, a worldwide survey of equine veterinarians found a median prevalence of PPID of 1% in equids in their practices, and only two‐thirds of PPID‐affected equids were receiving medications for PPID [7]. The discrepancy between the reported prevalence of PPID and the percentage of affected equids receiving treatment indicates that education of both veterinarians and equid owners is needed.

Approval of Prascend® (1 mg pergolide mesylate tablets, Boehringer‐Ingelheim Animal Health USA Inc., Duluth, GA) by the Food and Drug Administration in the fall of 2011 provided a consistent formulation of pergolide that was documented to improve both clinical signs and endocrine test results after 180 days of treatment in 86 of 113 (76%) of PPID‐affected horses and ponies [8]. Because PPID is considered a slowly progressive disorder, once started, pergolide is recommended as a life‐long daily treatment [9, 10, 11]. Before owners pursue long‐term medical treatment, they want to know how effective treatment might be and whether increases in medication dosage (and expense) will be required over years of treatment.

The purpose of our study was to characterize long‐term clinical response, endocrine test results, medical problems (potential adverse effects), outcome, and owner satisfaction in a cohort of equids enrolled in an extended use study of Prascend (pergolide tablets). Although the primary focus of the extended use study was to document the long‐term safety of pergolide, clinical responses and endocrine test results also were evaluated after 2.5, 3, 3.5, 4.5, 5.5, 6.5, 9.5, 12.5, and 14.5 years of treatment.

## 2 Materials and Methods

### 2.1 Animals

Thirty client‐owned equids that completed the 180‐day open field clinical efficacy study for Prascend® at Michigan State University were enrolled in an extended use study until death or euthanasia over a period of nearly 15 years. Enrollment in the initial efficacy study (day −7, from November 1, 2008 through January 31, 2009) required clinical signs of PPID (a composite clinical score was the sum of individual 0–3 scores assigned for hypertrichosis [a minimum score of 1 for hypertrichosis was required], hyperhidrosis, polyuria, polydipsia, abnormal fat deposition, and muscle wasting) and either supportive overnight dexamethasone suppression test results (ODST), with failure of suppression of endogenous cortisol concentration to < 1 μg/dL 19 h after IM administration of 40 μg/kg dexamethasone or an increased (> 50 pg/mL) plasma adrenocorticotropin (ACTH) concentration [8]. Equids in the efficacy study were started on Prascend® (pergolide tablets, 2 μg/kg PO q24h) and re‐evaluated by clinical examination and endocrine testing after 90 and 180 days. If endocrine test results remained abnormal (failure of an ODST or an ACTH concentration > 50 pg/mL) after 90 days, Prascend® dosage was increased to 4 μg/kg PO q24h, the maximum dose allowed in the study [8]. Equids in the extended use study were assigned to one of four groups based on endocrine test results after 90 and 180 days of the open field clinical efficacy study (pass [P] = normalization of endocrine test results and fail [F] = endocrine test results remained abnormal): (1) pass/pass (P/P, *n* = 13); (2) pass/fail (P/F, *n* = 4); (3) fail/pass (F/P, *n* = 4); and fail/fail (F/F, *n* = 9). Equids enrolled in the extended use study were maintained on the Prascend® dosage (2 [P/P] or 4 [P/F, F/P and F/F] μg/kg PO q24h) that the equids were receiving at the termination of the 180‐day efficacy study. The maximum Prascend® dose allowed in the extended use study remained 4 μg/kg PO q24h.

### 2.2 Assessment

Adherence to daily medication administration and medical problems (potential adverse effects) were documented by telephone interviews with owners every 3 months (as long as the equids survived). In addition, more serious medical problems and deaths were reported to study investigators by owners or primary care veterinarians as problems occurred. During telephone interviews, owners also were asked whether or not they were satisfied with their equid's current condition. Completion of telephone interviews was required before owners were shipped the next 3‐month supply of Prascend. In addition, after 2.5, 3, 3.5, 4.5, 5.5, 6.5, 9.5, 12.5, and 14.5 years of treatment, most equids either returned to Michigan State University for re‐evaluation or were examined at their home stables by study investigators. Re‐evaluation included clinical examination and endocrine testing (ODST and measurement of plasma ACTH and serum insulin and glucose concentrations). Unfortunately, not all animals were evaluated at all time points and not all endocrine tests were performed at each evaluation. All procedures performed were approved by the Animal Care and Use Committee of Michigan State University.

### 2.3 Laboratory Analyses

From the study start until the end of 2014, plasma cortisol concentration was determined using a commercially available solid‐phase radioimmunoassay (RIA; Coat‐A‐Count cortisol RIA, Diagnostic Products Corp. Los Angeles, CA) as previously described [12]. From 2015 onward, cortisol concentration was determined using a chemiluminescent immunoassay using the Immulite 2000XPi Immunoassay System (Siemens Medical Solutions, Malvern, PA). Assay of equine plasma ACTH concentration for the open field clinical efficacy study was performed using an immunoradiometric assay (IRMA) for ACTH_1–39_ (ACTH IRMA, DiaSorin, Stillwater MN), using the laboratory's reference interval of 9–45 pg/mL and validated for equine plasma as previously described [13]. For all follow‐up assessments, plasma ACTH concentration was measured using another IRMA (Scantibodies ACTH IRMA, Scantibodies Laboratory, Santee, CA), using the same laboratory reference interval of 9–45 pg/mL and validated for equine plasma, as previously described [14]. In 2021, the Michigan State University Veterinary Diagnostic Laboratory (MSU VDL) changed to the Immulite Immunoassay System (Siemens Medical Solutions, Malvern, PA) for measurement of plasma ACTH concentration and a normal result was < 35 pg/mL. Serum insulin concentration was measured in the open field clinical efficacy study using a commercially available RIA (Diagnostic Systems Laboratory, Webster, TX) that had been used in previous studies of equids [15]. For all follow‐up assessments, serum insulin concentration was measured using a human insulin specific RIA kit (MilliporeSigma, Burlington, MA) as previously described [16]. All assays were performed with duplicate tubes for standards and samples according to manufacturers' protocols with regard to sample volume and incubation times. These samples were submitted as routine clinical samples to the Endocrinology Laboratory at the Michigan State University Veterinary Diagnostic Laboratory (MSU VDL). Complete blood counts and serum biochemical profiles were similarly submitted as routine clinical samples to the Clinical Pathology Laboratory at the MSU VDL. When blood glucose concentration was the only biochemical variable measured at re‐evaluation, a glucometer was used (ACCU‐CHEK Performa, Roche Diabetes Care, Indianapolis, IN).

### 2.4 Interim Analysis of Factors Affecting Survival

To investigate factors that might predict long‐term survival, clinical signs and endocrine test results in the 180‐day open field clinical efficacy study were compared after 6 years of the extended use study between 20 equids that had either died (5) or had been euthanized (15) and the 10 surviving equids.

### 2.5 Owner Survey of Satisfaction With Long‐Term Treatment

Approximately10 years after enrollment, owners were asked to complete an on‐line survey to document their satisfaction with treatment, their willingness to treat another PPID‐affected equid with pergolide, and the annual expenditure they would be willing to pay for long‐term treatment.

### 2.6 Data Analysis

Descriptive statistics (mean ± SD or median and interquartile range [IQR]) were used to characterize clinical response and endocrine test results of the cohort enrolled in the extended use study. Normality was determined using the Kolmogorov–Smirnov test. Comparison of proportions of equids considered treatment successes, as well as percentage improvement in composite clinical scores between the entire cohort enrolled in the field efficacy study and the subgroup cohort enrolled in the extended use study were performed using *t* tests and *z* tests or a Mann–Whitney rank sum test. These tests also were used to compare data between equids with and without laminitis. Survival analysis of the four subgroups in the cohort (P/P, P/F, F/P, and F/F) was compared using a Gehan‐Breslow–Wilcoxon test. Clinical and laboratory data between the 10 survivors and 20 nonsurvivors after 6 years of the study were compared by one‐way analysis of variance (ANOVA) or one‐way ANOVA on ranks. Statistical analyses were performed using SigmaStat (Systat Software, San Jose, CA) and GraphPad Prism (GraphPad Software, Boston, MA). Significance was set at *p* < 0.05.

## 3 Results

### 3.1 Animals

The extended use study included 28 horses and two ponies and consisted of 17 geldings and 13 mares. Estimated mean age at enrollment was 23.1 years (range, 17–29) and breeds included nine Arabians or Arabian crosses, six Morgans or Morgan crosses, six Quarterhorses or Quarterhorse crosses, two Thoroughbreds, two ponies, one Tennessee Walking Horse, one Missouri Fox Trotter, one Paso Fino, one Fjord, and one mixed breed horse.

At enrollment in the open field clinical efficacy study, 28 equids had abnormal ODST results (with five also having a plasma ACTH concentration > 50 pg/mL) and two had increased plasma ACTH concentrations (65 and 123 pg/mL, both with normal ODST results). After completion of the 180‐day efficacy study, all equids were reported to have improved attitudes and hair coat shedding. Median composite clinical score decreased from 5.5 (day −7) to 2.0 (day 180) in the extended study cohort, as compared to a decrease from 4.9 to 1.5 for the cohort of 111 horses that completed the open field clinical efficacy study [8]. Percentage improvement in composite score was not different (*p* = 0.22) between these cohorts. Treatment success in the efficacy study was defined as normalization of ODST results (cortisol concentration < 1.0 μg/dL 19 h after dexamethasone administration) or a decrease in ACTH concentration (by at least 50% or to < 50 pg/mL) and improvement by > 1 in any clinical score without worsening of any other clinical score [8]. In the open field study cohort, 86/113 (76%) and, in the extended study cohort, 17/30 (57%) met this definition of treatment success (*p* = 0.07). Thus, initial clinical improvement after 180 days of treatment with Prascend (pergolide tablets) was similar in the subgroup of equids enrolled in the extended use study, as compared to the entire cohort of equids in the clinical efficacy study (Supporting Information File 1).

### 3.2 Compliance and Medical Problems Reported During Telephone Interviews

Compliance with medication administration was high, with all owners reporting daily administration of Prascend® for > 95% of treatment days. One owner prolonged the dosing interval to 48 h several times during the study period when a decrease in appetite occurred. Several medical problems (potential adverse effects) developed over the 14‐year study period (Table 1). Common problems included gastrointestinal disturbances (colic, diarrhea), loss of body condition (largely attributed to age and dentition), laminitis, arthritis, and worsening of hypertrichosis. Only one apparently new case of laminitis developed during the extended use study, although this equid could have had previously unrecognized laminitis. In another two equids, no exacerbation of historical laminitis reported at enrollment in the open field clinical efficacy was recognized during the extended use study (surviving 3.3 and 8.2 years). A decrease in appetite, the most common adverse effect reported in the open field clinical efficacy study [8], was reported for a similar percentage (33%) of equids in the extended use study, as compared to the efficacy study (also 33%). None of these medical problems, other than decreased appetite, could be attributed specifically to medication administration. In the clinical efficacy study, a decrease in appetite most often was reported with the onset of Prascend® treatment. However, equids enrolled in the extended use study had already been on treatment for 180 days, and episodes of decreased appetite varied in both duration and magnitude and were not associated with an increase in pergolide dosage.

**TABLE 1:** Medical problems reported and causes of death or euthanasia over a 12‐year treatment period in 30 equids with pituitary pars intermedia dysfunction.

Reported problem/adverse effect	# cases	% equids affected
Muscle wasting/weight loss	13	43
Abnormal shedding/↑hypertrichosis	13	43
Colic	11	37
Musculoskeletal pain/arthritis	10	33
Decreased appetite	9	30
Laminitis/sole abscess	9	30
Diarrhea/loose feces	8	27
Behavior change	5	17
Eye problem	4	13
Neurological disorder (cause undetermined)	3	10
Injury	3	10
Skin disorder	3	10
Choke	2	7
Pneumonia	2	7
Asthma	2	7
PU/PD (worsening)	1	3
Fracture	1	3
Epistaxis	1	3
Mass under tail (melanoma)	1	3
Causes of death/euthanasia		
Poor condition/wasting	6	20
Colic	5	17
Laminitis – attributed to PPID	5	17
Sudden death	4	14
Arthritis	4	14
Neurological disease (cause undetermined)	4	14
Fracture	1	3

### 3.3 Outcome

**FIGURE 1:**
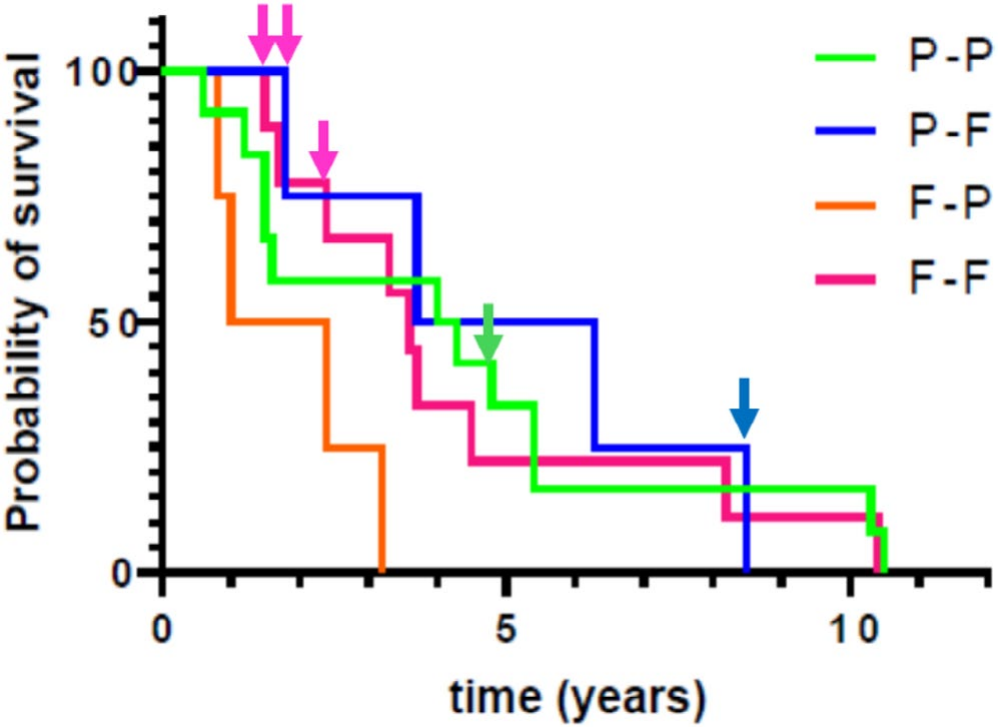
Kaplan–Meier survival analysis of time to death (*n* = 5) or euthanasia (*n* = 24) of 29 of 30 equids enrolled in an extended use study of Prascend® (pergolide tablets), for treatment of pituitary pars intermedia dysfunction. Survival curves for the four groups at the start of the extended pergolide treatment study (pass/pass [*n* = 12]; pass/fail [*n* = 4]; fail/pass [*n* = 4]; and fail/fail [*n* = 9]) are in different colors. There was no difference (*p* = 0.10) in survival between groups. Inverted arrows indicate the five horses euthanized due to chronic laminitis.

Over the course of the extended use study, five of 30 equids died (four sudden deaths and one within hours of onset of an acute neurological disorder) and 24 were euthanized between 0.6 and 10.5 years after onset of treatment. Median survival time was 3.6 years (IQR, 1.6–5.4 years) and was not different (*p* = 0.10) for the four groups (P/P, 4.2 years; P/F, 5.0 years; F/P, 1.7 years; and F/F, 3.6 years; Figure 1). At the time of manuscript submission, only one equid (a pony) remains alive. Euthanasia was performed because of complications of PPID, specifically laminitis, in five equids, whereas euthanasia of the remaining 19 equids was performed for disorders associated with advanced age (poor dentition and wasting [*n* = 6]), acute colic [*n* = 5], moderate to severe arthritis [*n* = 4], undiagnosed neurological disorders [*n* = 3], and (catastrophic forelimb fracture after falling on ice [*n* = 1]). Median survival time for horses with owner‐reported lameness or lameness consistent with laminitis (*n* = 9; 3.5 years; range, 1.0–8.5 years) was not different (*p* = 0.31) than median survival time for apparently nonlaminitic horses (*n* = 21; 3.6 years; range, 0.6–10.5 years).

### 3.4 Clinical Assessment and Endocrine Test Results

All owners reported sustained clinical improvement after 2.5 years of treatment (April, 2011; composite clinical scores were not performed at that time) and ODST results were normal in 75% of equids (18 of 24 horses, three were not tested at this time point and three had died; Table 2). Of interest, seven of 13 horses with abnormal OSDT results at the start of the extended use study (180‐day timepoint of open field clinical efficacy study) had normal ODST results after an additional 2 years of treatment without an increase in pergolide dose. Two horses with normal ODST results after 180 days of treatment had now failed the ODST and four horses had persistently abnormal ODST results.

**TABLE 2:** Overnight dexamethasone suppression test results (pass/fail/not tested) in 30 equids with pituitary pars intermedia dysfunction treated with Prascend® for up to 12.5 years.

90 and 180 day results	2.5 years	3.0 years	3.5 years	4.5 years	5.5 years	6.5 years	9.5 years	12.5 years
	Spring 2011	Fall 2011	Spring 2012	Spring 2013	Spring 2014	Spring 2015	Spring 2018	Spring 2021
# deaths	3	5	6	12	19	21	24	29
Pass/pass—13	10/1/1	3/5/2	8/1/1	5/3/0	5/1/0	4/1/0	2/1/0	1/0/0
Pass/fail—4	3/0/1	1/1/2	3/1/0	2/1/0	2/0/0	0/1/1	0/1/0	0/0/0
Fail/pass—4	1/1/0	0/2/0	1/1/0	0/1/0	0/0/0	0/0/0	0/0/0	0/0/0
Fail/fail—9	4/4/1	3/4/2	7/1/0	5/1/0	1/2/0	0/2/0	0/2/0	0/0/0
Total pass/fail/NT^a^	18/6/3	7/12/6	19/4/1	12/6/0	8/3/0	4/4/1	2/4/0	1/0/0

^a^ NT = not tested.

After 3 years of treatment (September–October, 2011), all owners remained satisfied with clinical improvement, but endocrine test results (all ODST) were normal in only 37% of horses (seven of 19 horses, six were not tested at this time point and two more had died). After 3.5 years of treatment (April, 2012), all owners continued to be satisfied with clinical improvement, and ODST results were normal in 83% of horses (19 of 23 horses, one was not tested and one more horse had died). After 4.5 years of treatment (February, 2013), all owners remained satisfied with clinical improvement. Of the 18 remaining equids (six additional horses had died), ODST results remained normal in 67% of horses (12/18 equids). The dosage of pergolide was increased to 4 μg/kg PO q24h in three equids in the P/P group at this time based on a failed ODST. Plasma ACTH concentration at this time point was ≤ 45 pg/mL (upper limit of laboratory reference interval for Scantibodies IRMA) in 12/17 (71%) equids (ACTH was not measured in one horse). Of the five horses with plasma ACTH concentrations > 45 pg/mL, results ranged from 58 to 91 pg/mL, and three of these horses had normal ODST results.

Although visual assessment confirmed that many equids had aged since enrollment in the extended use study (e.g., loss of muscle mass, swayback appearance), owners remained satisfied with the equids' attitude and overall condition after 5.5 years of treatment (May, 2014). Of the 11 remaining equids (seven additional horses had died), ODST results remained normal in 73% (8/11 equids) and plasma ACTH concentration was ≤ 45 pg/mL in seven of these animals. Two of four horses with plasma ACTH concentrations > 45 pg/mL (53 and 59 pg/mL) had normal ODST results, whereas the other two had only abnormal ACTH results (49 and 92 pg/mL). Two additional horses died over the next year, decreasing the surviving cohort to nine equids after 6.5 years of treatment (May, 2015). Eight of nine equids were tested at this time point: four failed the ODST (two had plasma ACTH concentrations > 45 pg/mL [77 and 79 pg/mL]) and four had normal ODST results, with three having plasma ACTH concentrations > 45 pg/mL (54, 62, and 66 pg/mL).

After 9.5 years of treatment (April, 2018) the cohort had decreased to six equids (P/P [*n* = 3]; P/F [*n* = 1]; F/P [*n* = 0]; F/F [*n* = 2]) with two of three P/P equids continuing to have normal ODST results on a dosage of 2 μg/kg PO q24h (with plasma ACTH concentrations of 34 and 104 pg/mL). The other four horses had abnormal ODST results and plasma ACTH concentrations ranging from 45 to 164 pg/mL. The one surviving pony (on 2 μg/kg PO q24h) had a normal plasma ACTH concentration (32 pg/mL) in April 2021 but ACTH had increased to 47 pg/mL by May 2023 (using a reference interval of < 35 pg/mL for the Immulite Immunoassay system).

Basal serum insulin concentration (samples collected within 2 h after hospital admission without feed, and after variable duration of transport) was increased (> 300 pmol/L) in 12 equids at the start of the clinical efficacy study (day −7) and six had laminitis. In the six equids with hyperinsulinemia and laminitis, insulin concentration (median, 618 pmol/L; IQR, 545–2153) was not different (*p* = 0.33) than in the six hyperinsulinemic horses without laminitis (median, 686 pmol/L; IQR, 472–919; Supporting Information File 1). However, for the nine horses with laminitis (eight horses with evidence of chronic laminitis at the start of the clinical efficacy study [day −7] and one horse that developed laminitis during the extended use study) median insulin concentration (545 pmol/L; IQR, 391–103) was higher (*p* < 0.03) than in the 21 nonlaminitic horses (median, 194 pmol/L; IQR, 128–284). Insulin concentration was measured again after 90 and 180 days of the clinical efficacy study and after 3.5, 5.5 and 9.5 years of the extended use study. After 180 days of the clinical efficacy study, six equids had an increased insulin concentrations with only one having laminitis. The other five equids with hyperinsulinemia did not have laminitis. However, insulin concentration had decreased (*p* < 0.01) in the laminitic group (median, 276 pmol/L; IQR, 112–285) and was not different (*p* = 1.0) from the nonlaminitic group (median, 170 pmol/L; IQR, 78–258). Differences in basal insulin concentration between laminitic and nonlaminitic equids were not detected throughout the remainder of the extended use study.

At the beginning of the clinical efficacy study, three horses had hyperglycemia (130–148 mg/dL; reference interval, 78–124) and five horses had hypertriglyceridemia (76–160 mg/dL; reference interval, 9–71). After 180 days of pergolide treatment, blood glucose concentration remained increased (214 mg/dL) in only one of the three horses that had hyperglycemia on day‐7, and only two of the initial four horses with hypertriglyceridemia had persistently increased triglyceride concentrations (108 and 149 mg/dL). After 4.5, 5.5, and 9.5 years, only one horse had hyperglycemia (228 mg/dL at 4.5 years compared to 214 mg/dL at day 180; no additional glucose concentrations were measured in this horse). This horse also had moderate hypertriglyceridemia (522 mg/dL) at 4.5 years, compared with 149 mg/dL at day 180.

### 3.5 Interim Analysis of Factors Affecting Survival

After 6 years of the extended use study, clinical data and endocrine test results at enrollment (day −7) and after the initial 180 days of treatment with Prascend® of the clinical efficacy study were compared between 20 equids that had either died (5) or been euthanized (15) and the 10 surviving equids. The only significant difference between survivors and nonsurvivors was cortisol concentration 19 h after administration of dexamethasone with the ODST on day −7 (Table 3).

**TABLE 3:** Comparison of mean ± S.D. or median, IQR for variables measured at days −7 and 180 of the open field clinical efficacy study for Prascend® (pergolide tablets) between groups of horses that survived (*n* = 10) or failed to survive (*n* = 20) after the initial 6 years of the extended use study.

Variable	Survivors (*n* = 10)	Nonsurvivors (*n* = 20)	*p*
Age (years, many estimated)	21.6 ± 3.7	23.8 ± 3.9	0.15
Sex	7 G/3 F	10 G/10 F	0.51
180‐day success rate	60%	55%	0.90
Composite clinical score, day −7	4.9 ± 2.0	6.2 ± 2.8	0.22
Composite clinical score, day 180	2.2 ± 1.5	2.8 ± 1.6	0.37
Change in composite clinical score	2.7 ± 1.6	3.4 ± 1.9	0.32
Laminitis, day −7	30%^a^	25%^a^	0.89
Laminitis, day 180	30%^a^	25%^a^	0.89
Cortisol (μg/dL), predexamethasone, day −7	4.9, 2.9–7.6	5.6, 4.3–7.7	0.55
Cortisol (μg/dL), postdexamethasone, day −7	2.9, 2.2–3.6	3.9, 2.7–4.8	**0.04**
% suppression	36 ± 46	21 ± 44	0.39
Cortisol (μg/dL), predexamethasone, day 180	10.1 ± 5.0	8.1 ± 3.1	0.21
Cortisol (μg/dL), postdexamethasone, day 180	0.8, 0.4–1.2	0.8, 0.4–3.0	0.53
% suppression	89, 86–96	86, 68–95	0.20
ACTH (pg/mL), day −7	34, 31–82	39, 23–48	0.93
Insulin (μU/mL), day −7	30, 19–64	37, 21–83	0.40
Insulin (μU/mL), day 180	32, 17–40	25, 10–44	0.74
Glucose (mg/dL), day −7	94, 86–100	98, 90–103	0.30
Glucose (mg/dL), day 180	101, 98–109	96, 90–107	0.25
Triglycerides (mg/dL), day −7	35, 18–42	36, 25–63	0.50
Triglycerides (mg/dL), day 180	33, 17–40	26, 18–41	0.79

*Note:* Bold value indicates statistically significant results (*p* < 0.05).

^a^ Same horses in each group with laminitis at day −7 and day 180.

### 3.6 Owner Survey

Of the 29 equid owners contacted (one owner had two horses in the study), 25 (86%) completed the survey. Seventy‐one percent and 70% strongly agreed and 25% and 30% agreed that treatment with Prascend improved their perception of the equid's quality of life and prolonged lifespan, respectively. Improvement in clinical signs was most satisfactory for energy level (77%), hair coat (71%), and muscle mass (61%). Overall satisfaction with treatment on a scale of 1–10 (10 best) was 5: 9%, 7: 9%, 8: 13%, 9: 17%, and 10: 52%, and 87% of owners either agreed or strongly agreed that they would provide lifelong treatment if they had another equid with PPID. However, medication cost would be a factor: 26% and 57% of owners were willing to pay an annual cost of $500 or $1000, respectively, whereas only 17% of owners would be willing to pay ≥$1,500 annually.

## 4 Discussion

Our results show that long‐term (> 5 years) treatment of PPID‐affected equids with pergolide mesylate produces clinical improvement in nearly all surviving equids, and normalization of endocrine test results in > 60% of cases. Furthermore, owner satisfaction was high. When asked if they would consider treatment of another PPID‐affected equid with pergolide, owners who participated in the extended use study responded affirmatively, similar to a previous report [17]. Although all owners responding to the survey thought that treatment with Prascend® prolonged their equid's lifespan and improved quality of life, our study did not produce data to support this claim because a cohort of untreated PPID‐affected equids was not followed over the same time period. Median survival time (3.6 years; IQR, 1.6–5.4 years) after study enrollment was comparable to the survival of 14/29 equids with PPID for 4.6 years after diagnosis in a previous report [17]. In that study, 25% of equids remained alive after 5.3 years, despite inconsistent treatment and use of compounded pergolide products with or without cyproheptadine. Other studies in Switzerland [18] and Australia [19] reported clinical improvement in 79% and 64% of pergolide‐treated equids with median follow‐up times of 8 and 11 months, respectively. At the time of follow‐up (median, 11 months; range 0–85 months) in the Australian cohort of 191 equids, pergolide treatment was more likely to result in survival (95%) than no treatment (81%), suggesting a potential life‐extending effect of pergolide treatment [19]. In contrast, an older study reported a shorter mean survival time of 162 days (range, 86–368 days) after diagnosis of PPID without medical treatment [20]. In our extended use study, euthanasia of five horses with laminitis could be attributed to PPID whereas the remaining 24 equids died or were euthanized for problems associated with advancing age (e.g., arthritis, loss of condition, or colic due to strangulating lipoma) rather than PPID, as has been reported in other studies of mortality of aged equids [21, 22].

During the 180‐day clinical efficacy study for Prascend, 86/113 (76%) of enrolled equids were considered treatment successes [8]. Improvement in clinical signs (89% had a decrease in hypertrichosis score) was more common than normalization of endocrine test results (58%). This discrepancy was similar to previous studies that reported improvement in clinical signs with the persistence of abnormal endocrine test results [17, 18, 19, 23, 24, 25, 26]. Furthermore, clinical signs that are more difficult to quantify (e.g., attitude, energy level) were anecdotally reported to improve more rapidly than changes in haircoat and muscle mass, which required several months to recognize improvement. In fact, a few owners reported their equids to “wake up” and have increased activity within the initial 1–2 weeks of treatment.

During the extended use study, > 60% of the 13 equids in the P/P group continued to have normal ODST results for 5–6 years without an increase in pergolide dosage. To our knowledge, our study is the first long‐term treatment study to provide evidence that equids may continue to respond favorably to the initial pergolide dose, rather than requiring an increase in dose over time. This finding should be of interest to primary care veterinarians counseling owners about lifelong medical treatment of PPID‐affected equids. Surprisingly, ODST results also normalized for 4–5 years in > 50% of equids in the F/F group (that had failed the 180‐day ODST and had a dosage increase to 4 μg/kg after the initial 90 days of the clinical efficacy study). Finally, in all groups the highest percentage (> 60%) of abnormal ODST results was found after 3 years of treatment in the fall of 2012. This finding suggests that increased seasonal activity of the hypothalamic–pituitary–adrenal axis persists in fall months despite treatment with pergolide.

Medical problems (potential adverse effects of pergolide) observed in our study were both similar to and different from adverse reactions reported in the open field clinical efficacy study (Supporting Information File 1). First, a transient decrease in appetite was reported in 33% of equids in both studies [8]. Unlike the clinical efficacy study, changes in appetite varied in severity and duration in the extended use study and were unrelated to starting or changing the dose of Prascend. Thus, it is uncertain whether decreases in appetite were associated with the drug or simply a problem that can wax and wane in aging equids [6]. Three medical problems observed with frequencies of 30%–43% could be associated with PPID: (1) laminitis and sole abscess; (2) muscle wasting and weight loss, and (3) abnormal shedding and progression of hypertrichosis. Intermittent exacerbations and failure to effectively control laminitis pain contributed to a decision for euthanasia in five horses, four of which had advanced signs of PPID (hypertrichosis scores of 3) and all five had increased serum insulin concentrations at the start of the study. The relationship between PPID and laminitis remains incompletely understood although concurrent insulin dysregulation (ID) is a known risk factor for laminitis in PPID‐affected equids [18, 27]. A recent study assessing insulin sensitivity, using a combined glucose and insulin tolerance test (CGIT), found no differences in CGIT variables between equids with PPID and ID as compared to equids with ID alone after 4 weeks of treatment with pergolide [28]. However, both peak insulin response and area under the insulin curve after a starch meal were attenuated in the PPID and ID group, but not the ID alone group, after pergolide treatment [28]. Although serum insulin concentration also was observed to decrease after 180 days of pergolide treatment in the clinical efficacy study [8], evidence for a direct effect of pergolide on glucose and insulin dynamics is lacking. Results of the extended use study further support that pergolide should not be considered a primary treatment for laminitis, but clinical improvement with pergolide treatment of PPID‐affected equids might decrease the severity of laminitis pain and prolong the interval between exacerbations. Although muscle wasting is not a specific sign of PPID (because it can be observed with advancing age [6]) clients reported this problem in 33% of the equids in our study. Finally, abnormal shedding and progressive hypertrichosis [29] are pathognomonic signs of PPID, and progression of hair coat changes, rather than abnormal ODST results, was the reason for an increase in pergolide dose in two horses in the P/P group. Muscle wasting and haircoat abnormalities became more common as the extended study continued beyond 5 years, when aging became apparent.

Gastrointestinal disorders including colic and episodes of diarrhea or unformed feces were reported in approximately one‐third of the equids, with acute colic the reason for euthanasia of five horses. Two of these animals had necropsy examinations that identified a strangulating lipoma, a problem not considered an adverse effect of pergolide. Diarrhea also was reported as a potential adverse effect of pergolide in 10% of equids in the clinical efficacy study. In the extended use study, episodes of diarrhea were usually self‐limiting, and owners managed this problem using diet changes or administration of probiotics or psyllium products for possible sand accumulation. A few less common medical problems (Table 1) were not considered related to pergolide treatment.

An interim comparison of clinical signs and laboratory results that might predict survival vs. nonsurvival was performed approximately half‐way through the extended study, but no specific risk factors were identified. The only significant finding was that nonsurvivors (*n* = 20) had higher cortisol concentrations than survivors (*n* = 10) 19 h after dexamethasone administration on day −7 of the clinical efficacy study. Although not significantly different, percentage suppression of endogenous cortisol concentration during the day −7 ODST was numerically less (21%) for nonsurvivors compared with survivors (36%). This finding provides weak evidence that nonsurvivors could have had more severe disease than survivors at enrollment in the open field clinical efficacy study.

The survey of equid owners after 10 years into the extended use study had a high response rate, although recall bias could have affected responses because many equids had been dead for several years when surveys were completed. Nevertheless, the surveys provided valuable information. Nearly all (96%) owners agreed that treatment with Prascend® improved their equids' quality of life, and 87% agreed that they would provide lifelong treatment if they had another equid with PPID. However, medication cost would be a factor in their decision for treatment. In the extended use study, treatment compliance was high and can be attributed to the requirement that owners complete quarterly interviews to receive the next 3‐month supply of Prascend® at no cost. In a study of treatment compliance in 110 equids with PPID, in which owners had to pay for the medication, only 48% were compliant in administering ≥ 90% of the veterinarian‐recommended dose of pergolide to their equids [30].

Our study had several limitations. First, the ODST, rather than plasma ACTH concentration, was the primary endocrine test performed to both diagnose and monitor PPID. The ODST was selected because of knowledge about PPID diagnosis at the time the clinical efficacy study was designed. However, ODST results might not parallel changes in ACTH concentration. Second, different hormone assays were used during the study, and results, particularly for ACTH, might not be comparable. Third, clinical scoring was not performed, and not all hormones were measured at all follow‐up assessments. Fourth, both recall and receiving Prascend at no cost might have biased survey responses. Finally, a placebo‐treated group was not studied in parallel to the equids receiving Prascend.

In conclusion, long‐term treatment with pergolide mesylate improved the quality of life of PPID‐affected equids, leading to high owner satisfaction. Our findings complement published recommendations for veterinarians advising owners about the medical treatment of PPID‐affected equids [31]. However, veterinarians should be cautious in suggesting that pergolide treatment might prolong life, because the cause of death or decision for euthanasia was unrelated to PPID in most equids lost during this study.

## Disclosure

Authors declare no off‐label use of antimicrobials.

## Ethics Statement

Approved by the Animal Care and Use Committee of Michigan State University (approval 11/08‐191‐00). Authors declare human ethics approval was not needed.

## Conflicts of Interest

Dr. Schott has received research funding from and has been a consultant to Boehringer‐Ingelheim Animal Health USA. Dr. Schott has also received travel support from Boehringer‐Ingelheim Animal Health USA. The other authors declare no conflicts of interest.

## Supporting information


**Data S1** Supporting Information.
